# Food Safety Knowledge, Attitude, and Practices (KAP) of Urban Consumers in Low-Income and Lower-Middle-Income Countries (LLMICs): A Scoping Review

**DOI:** 10.3390/foods15081381

**Published:** 2026-04-16

**Authors:** Samira Choudhury, Antonieta Medina-Lara, Afrin Zainab Bi, Phoebe Ricarte, Nia Morrish, Prakashan C. Veettil

**Affiliations:** 1 International Rice Research Institute (IRRI), Pili Drive, Los Baños 4031, Laguna, Philippines; p.ricarte@cgiar.org; 2 Public Health Economics Group, Department of Public Health and Sport Sciences, University of Exeter Medical School, University of Exeter, B3183, Exeter EX1 2HZ, UK; a.medina-lara@exeter.ac.uk (A.M.-L.); n.g.morrish@exeter.ac.uk (N.M.); 3 International Rice Research Institute (IRRI), South Asia Regional Centre, NSRTC Campus, Varanasi 221106, India; a.zainab@cgiar.org; 4 International Rice Research Institute (IRRI), 1st Floor, CG Block, NASC Complex, Dev Prakash Shastri Marg, Pusa, New Delhi, Delhi 110012, India; pc.veettil@cgiar.org

**Keywords:** food safety, consumers, knowledge, attitude, practices, scoping review

## Abstract

Food safety is a major global public health concern and a key contributor to the burden of foodborne diseases. This scoping review examined the knowledge, attitudes, and practices (KAP) related to food safety among urban consumers in low- and lower-middle-income countries (LLMICs). A systematic search was conducted across seven electronic databases: Medline (PubMed), Web of Science (Social Science Citation Index), Embase (Ovid), Global Health (Ovid), PsycINFO (Ovid), Econlit (EBSCOhost), and Scopus to identify studies published in English between 2000 and 2025. Data extraction and quality appraisal were conducted independently by two reviewers, and findings were synthesized in a narrative analysis. Twenty-six studies from 14 LLMICs met the inclusion criteria. Of the 25 studies assessing knowledge and awareness, the majority reported that consumers had some understanding of food safety, although 10 (40%) highlighted limited awareness. Fifteen studies examined practices, with several noting appropriate behaviours; however, nine (56.2%) reported poor practices. Seven studies assessed attitudes, with most reflecting positive perceptions, while one (16.7%) identified negative views. Only four studies examined the full KAP triad. Across studies, factors such as age, education, gender, marital status, training, employment status, income, field of study, and residential status were found to influence food safety KAP. Overall, the evidence suggests that while consumers in urban LLMIC settings generally demonstrate some knowledge and positive attitudes towards food safety, there remain significant gaps in practices that could compromise public health. Future research should prioritise underrepresented regions, employ more rigorous study designs, and incorporate longitudinal and qualitative approaches to gain deeper insights and inform targeted interventions.

## 1. Introduction

Globally, food safety is a critical public health issue contributing to various foodborne diseases. Foodborne diseases are illnesses caused by ingesting food or beverages contaminated with biological (such as bacteria, viruses, or parasites), chemical (such as toxins or pesticide residues), or physical (such as foreign objects) hazards.

Foodborne diseases are a serious public health threat. According to the World Health Organization (WHO) [1] estimates, there are 600 million cases of foodborne diseases and 42 million related deaths every year, a health burden comparable to that of malaria, HIV/AIDs, or tuberculosis [2,3]. The burden of foodborne diseases falls disproportionately on populations in low- and middle-income countries (LMICs) [4,5] which account for 75% of global foodborne disease-related deaths, despite representing only 41% of the world’s population. The economic impact in these settings is substantial, with annual costs estimated to exceed US$100 billion per year [3]. In addition, globally, foodborne diseases contribute an estimated 33 million Disability Adjusted Life Years (DALYs) [2].

Preventing foodborne disease is complex, involving different actors across the entire food system [6]. Although improper food handling and storage at the household level are well-recognized risk factors, contamination can occur at multiple points along the food supply chain before foods reach consumers. Evidence shows that many foods, both fresh and ready-to-eat, sold in traditional markets with poor market infrastructure [7], unsanitary conditions [8,9], and inadequate storage practices by vendors [10] are already contaminated at the point of purchase [11].

In contrast to high-income countries, many LMICs face substantial challenges due to limited government capacity, insufficient funding, inadequate infrastructure, and socioeconomic disparities that hinder the implementation of effective food safety regulation, inspection, training, and testing systems [4,12]. These challenges are compounded by the limited access of regulatory bodies to traditional food markets where most consumers purchase their food [13,14], further contributing to the high incidence and economic burden of foodborne diseases in these regions [15,16].

While home-cooked meals are generally regarded as safer in LMICs [17], urbanization and rising incomes are increasing demand for food purchased from markets or consumed outside the home [13,18,19]. The growing popularity of street foods, driven by affordability, accessibility, and convenience, also poses risks where food safety regulations and controls are weak or poorly enforced [20,21,22]. Consequently, reducing foodborne disease risk is an urgent priority in LMICs.

In rapidly urbanizing LMIC settings, a significant share of foodborne illnesses is linked to the unsafe handling and preparation of fresh foods, particularly animal-based products and fresh fruits and vegetables [4,12,23,24]. Recent evidence underscores the growing urgency of addressing food safety within urban food systems. According to [25], 1.7 billion food-insecure individuals now live in urban areas. Food safety concerns are disproportionately concentrated in low-income urban and peri-urban areas. In these settings, food safety challenges arise from both individual-level constraints and broader systemic weaknesses. Given that 60–80% of food poisoning cases originate from household food handling practices [26,27,28,29], consumers play a pivotal role in ensuring food safety. The household represents the final barrier against foodborne disease transmission, but can also be a critical point of contamination spread [30,31]. Therefore, safe food handling and hygiene behaviours at the household level are essential in reducing foodborne diseases [32], with consumer knowledge, attitudes, and practices (KAP) being central to achieving this goal [6,29,33,34]. However, consumer food safety KAP can vary widely and is influenced by factors such as education, socioeconomic status, and cultural background. Therefore, a thorough understanding of these KAP dimensions among consumers is critical for designing effective interventions, education, and training programs that promote safe food handling, preparation, storage, and hygiene.

Although various reviews have explored food safety perspectives and practices of consumers at the country level [5,33,34], there remains a gap in synthesizing evidence on food safety KAP among consumers specifically in urban low-income and lower-middle income countries (LLMICs)—populations that bear a high burden of foodborne diseases and are key actors in ensuring household food safety. Understanding consumers’ KAP is fundamental to improving food safety in urban LLMICs, where systemic weaknesses heighten reliance on individual behaviours as a primary line of defence. To address this, the present scoping review consolidates existing research on consumer food safety KAP in urban LLMIC settings, addressing the following four research questions.

(1) What is the evidence on food safety KAP of consumers across urban LLMICs?
(2) What factors (economic and sociodemographic) are associated with food safety KAP of consumers?
(3) What methods have been used to assess food safety KAP of consumers?
(4) How can this research be strengthened in the future?

## 2. Methods

### 2.1. Scoping Review

Scoping reviews are particularly useful for synthesizing evidence from a heterogeneous body of literature that has not been previously systematically reviewed and for identifying gaps in this existing research [35,36]. This review adhered to the Preferred Reporting Items for Systematic Reviews and Meta-Analyses (PRISMA) extension for scoping reviews checklist and guidelines (Supplementary Table S3) [37,38]. It was aligned with the methodological framework proposed by Arksey and O’Malley [39], further developed by Levac et al. [40] to ensure a robust and replicable process. The review protocol was registered and published on Open Science Framework (OSF): https://doi.org/10.17605/OSF.IO/HBYJ6.

### 2.2. Search Strategy

A structured search was undertaken in seven electronic databases: Medline (PubMed), Web of Science (Social Science Citation Index), Embase (Ovid), Global Health (Ovid), PsycINFO (Ovid), Econlit (EBSCOhost), and Scopus on 10th of April 2025 using a syntax of keywords and subject headings. Preliminary scoping searches were conducted to refine the search strategy, ensuring relevant studies were identified with the search syntax. The final search syntax incorporated text words and indexing terms specific to the various databases (e.g., MeSH for Pubmed). The search syntax was first developed for PubMed then modified to the additional database-specific search requirements. In accordance with the review objectives, this scoping review was restricted to peer-reviewed literature and did not include grey literature sources.

The selection of databases was guided by the interdisciplinary and consumer-focused nature of food safety knowledge, attitudes, and practices research. Databases with broad coverage of public health, nutrition, behavioral science, and social science literature were prioritized, as these are the primary disciplines in which consumer-focused food safety KAP studies are published, including research from low- and lower-middle-income countries. Regional health databases, which primarily index clinical and biomedical literature, were not searched as they were considered unlikely to capture additional eligible studies aligned with the objectives of this review.

The search terms used in these databases were as follows: (adult[MeSH] OR adult* OR “general population” OR adolescent[MeSH] OR adolescent* OR consumer*) AND (“food safety” OR “food borne” OR foodborne OR “food handling” OR “food preparation” OR “food poisoning” OR “food hygiene” OR “safe food” OR “food contamination” OR “food packaging” OR “food storage” OR “food scare*” OR “food sanitation” OR “food quality” OR “food adulteration” OR “food inspection”) AND (“knowledge” OR “awareness” OR “belief∗” OR “attitude∗” OR “opinion∗” or Pract* or Priori* or Expect* OR “Knowledge, Attitude, and Practices”) (Supplementary File Table S1: Search strategy).

### 2.3. Inclusion Criteria and Exclusion Criteria

The eligibility criteria were defined using the Population, Concept, and Context (PCC) framework [41] (Table 1). Original, peer reviewed studies employing qualitative, quantitative, and mixed method designs were eligible for inclusion. Study populations had to include adolescents (10–18 years) or adults. Only full-length articles published in English from 2000 onwards were considered, as earlier studies were deemed less relevant given the substantial transformations in urban food systems in LLMICs over the past two decades. To be included, studies were required to address at least one element of the KAP elements related to food safety, be focused on consumers, and be conducted in urban settings within countries classified as low- and lower-middle-income countries (LLMICs) (using the World Bank definition of LMICs as of 2025) [42]. In this review, urban settings were defined according to the classification used in the original studies, which typically relied on national statistical definitions (e.g., cities, municipalities, metropolitan areas, or formally designated urban administrative units). An urban area is a densely populated and contiguous built-up settlement characterized by predominantly non-agricultural activities and higher levels of infrastructure and services, typically delineated using criteria such as population density, settlement size, and spatial contiguity [43]. Historically, countries have used diverse criteria, including population size, density, administrative status, and economic function, to distinguish urban from rural areas, reflecting national contexts and policy priorities. To enable global comparability, the UN Statistical Commission in 2020 endorsed the Degree of Urbanization, a harmonized methodology that classifies an entire national territory along an urban rural continuum by defining cities, towns, and rural areas [44].

Studies were excluded if they were any of the following: (1) carried out in upper middle-income countries; (2) reported specifically on the impacts of COVID-19; (3) did not meet the Population, Concept, and Context (PCC) framework requirements; (4) did not assess food safety KAP; (5) were not original peer-reviewed publications; (6) were non-English language publications; (7) were conducted in rural-only settings; (8) were reviews including systematic reviews, protocols, theses, conference proceedings/abstracts, news articles, case series or case reports.

### 2.4. Data Screening

Complete references were imported and de-duplicated in Endnote 20. Titles and abstracts were independently screened by two reviewers (SC and AZ) to identify articles with the potential to meet the inclusion criteria outlined above. Full texts were retrieved and again independently assessed for eligibility by the same two reviewers. Any disagreements were resolved through discussion between the two reviewers (SC and AZ) and with a third reviewer (PCV) where required. Reference lists of relevant reviews identified in database searches were checked to identify any additional eligible primary studies. Forward and backward citation chasing was conducted on studies included at full text.

### 2.5. Data Extraction

Relevant information was extracted from each of the included studies and placed into a standardized data form by the two reviewers (SC and PR). Extracted data included the following: author (year); country where the study was conducted; study population (age, sample size, gender, education status, economic status); study design (quantitative, qualitative, mixed method); and aspects assessed (KAP).

### 2.6. Quality Assessment

Two authors (SC and AZ) independently conducted quality appraisals of all included papers using the Mixed Methods Appraisal Tool (MMAT), 2018 version [45]. The MMAT was selected as it appraises the methodological quality of quantitative studies. Discrepancies in the ratings were discussed until agreement was reached. Each study was assessed for the appropriate criterion using a categorical scale as “yes” (1), “no” (2), “can’t tell” (3). MMAT scores representing the total number of “yes” responses were divided by the total number of criteria (five) and converted into percentages. Scores ranged from 20% (indicated *, low quality) to 100% (indicated *****, high quality). The appraisal scores were discussed by SC and AZ and in case of disagreements, the question was rated as “no” (2) or “can’t tell” (3). No overall quality score from the ratings of the included studies was calculated, as advised by [45]. Studies were not excluded based on the quality appraisal (Table 3: Quality assessment).

## 3. Results

### 3.1. Search Results

**Figure 1:**
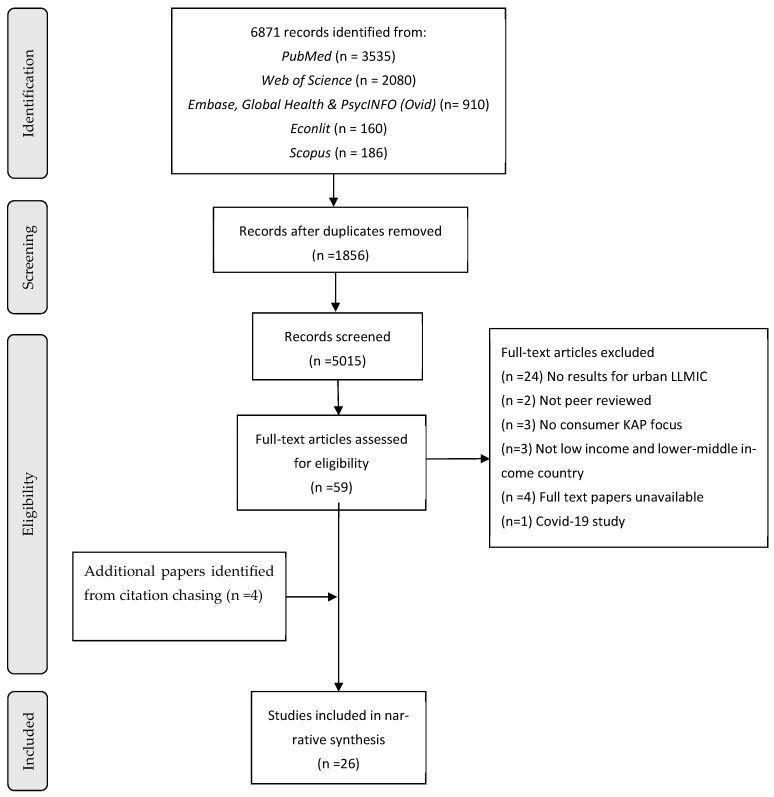
PRISMA diagram [46].

A total of 6871 records were initially retrieved through database searches. After removing duplicates, a total of 5015 records were screened by title and abstract applying initial screening criteria. Of these, 59 records met the initial inclusion criteria and were assessed for full-text eligibility. Following full-text review, 37 articles were excluded with documented reasons. A detailed list of studies excluded after full-text screening, along with the reasons for exclusion, is provided in Supplementary Table S3. Forward and backward citation chasing of the 22 included studies identified four additional studies that met the inclusion criteria and were subsequently included in the review. Therefore, in total, 26 studies were included in this review. Details of the study screening process are presented in a PRISMA flow diagram (Figure 1).

### 3.2. Characteristics of Included Studies

**Figure 2:**
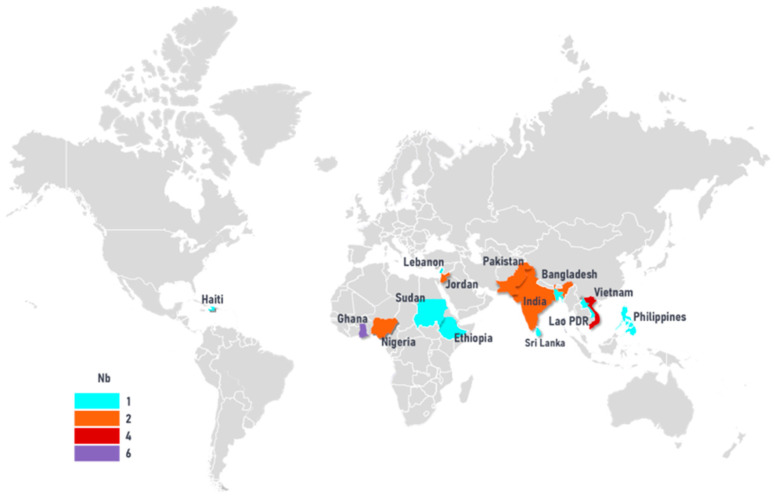
Map showing density of food safety research. Footnote Nb = number of studies conducted in each country.

All included studies were cross-sectional and published between 2008 and 2024. In total, the 26 studies were conducted across 14 different LLMICs (Figure 2). The majority (n = 24; 92%) were conducted in lower-middle-income countries (Ghana (n = 6), Vietnam (n = 4), Pakistan (n = 2), Nigeria (n = 2), Jordan (n = 2), India (n = 2), Lebanon, Bangladesh, Laos, Haiti, Sri Lanka, and Philippines. Only two (8%) studies were from low-income countries (Ethiopia, Sudan). There were no multi-country studies (See Table 2 for further details).

### 3.3. Quality Appraisal of Included Studies

The included studies implemented a range of outcome indicators, measures, and metrics. However, several methodological limitations were noted across the studies. Common issues included the following: lack of information on sampling strategy or sample representativeness; use of non-standardized, unvalidated, or untested instruments and methods; missing information on response rates and potential sources of bias; small or non-representative samples; unclear analytical methods. Overall, the quality of the research was generally low. Only eight studies were assessed to be of strong quality (≥80%), while four studies were rated as having moderate quality (≥60%) (Table 3). Quality scores were not used to include or exclude studies but rather to describe the quality of available evidence as part of the mapping component of this review.

**Table 3:** Critical appraisal of included sources of evidence using Mixed Methods Appraisal Tool (MMAT).

Publication	Quality	1.1–1.5	4.1	4.2	4.3	4.4	4.5	5.1–5.5
[47]	*	n/a	3	2	1	3	2	n/a
[48]	*	n/a	2	2	3	3	2	n/a
[49]	*	n/a	3	3	1	3	2	n/a
[50]	*	n/a	1	3	3	3	2	n/a
[51]	***	n/a	1	3	1	1	3	n/a
[52]	**	n/a	3	3	1	3	1	n/a
[53]	***	n/a	3	1	1	3	1	n/a
[54]	***	n/a	3	1	1	1	3	n/a
[55]	**	n/a	3	1	1	3	3	n/a
[56]	-	n/a	3	2	2	3	2	n/a
[57]	**	n/a	1	1	3	3	3	n/a
[58]	**	n/a	1	3	1	3	2	n/a
[59]	**	n/a	1	1	3	3	3	n/a
[60]	****	n/a	1	1	1	3	1	n/a
[61]	***	n/a	1	3	1	3	1	n/a
[62]	**	n/a	3	3	1	3	1	n/a
[63]	****	n/a	3	1	1	1	1	n/a
[64]	****	n/a	1	1	1	3	1	n/a
[65]	****	n/a	3	1	1	1	1	n/a
[66]	****	n/a	1	1	1	3	1	n/a
[67]	****	n/a	3	1	1	3	1	n/a
[68]	****	n/a	3	1	1	3	1	n/a
[69]	*	n/a	3	3	1	3	3	n/a
[70]	*****	n/a	1	1	1	1	1	n/a
[71]	**	n/a	3	3	1	3	1	n/a
[72]	*	n/a	2	3	3	3	1	n/a

^1^ MMAT checklist: Qualitative studies (1.1 Is the qualitative approach appropriate to answer the research question?, 1.2 Are the qualitative data collection methods adequate to address the research question?, 1.3 Are the findings adequately derived from the data?, 1.4 Is the interpretation of results sufficiently substantiated by data?, 1.5 Is there coherence between qualitative data sources, collection, analysis and interpretation?); Quantitative descriptive studies (4.1 Is the sampling strategy relevant to address the research question?, 4.2 Is the sample representative of the target population?, 4.3 Are the measurements appropriate?, 4.4 Is the risk of nonresponse bias low?, 4.5 Is the statistical analysis appropriate to answer the research question?); Mixed Methods studies (5.1 Is there an adequate rationale for using a mixed methods design to address the research question?, 5.2 Are the different components of the study effectively integrated to answer the research question?, 5.3 Are the outputs of the integration of qualitative and quantitative components adequately interpreted?, 5.4 Are divergences and inconsistencies between quantitative and qualitative results adequately addressed?, 5.5 Do the different components of the study adhere to the quality criteria of each tradition of the methods involved?). ^2^ Rating: 1 = yes, 2 = no, 3 = can’t tell. ^3^ Quality: ***** 100% criteria met, **** 80% criteria met, *** 60% criteria met, ** 40% criteria met, * 20% criteria met; dash (-) indicates no criteria are met. ^4^ Only peer-reviewed published articles appraised for quality (n = 26).

### 3.4. Design and Methods of Included Studies

All included studies exclusively employed quantitative descriptive methods, either to describe or examine indicators related to food safety KAP, or to assess their associations with relevant outcomes of interest. Each study used a cross-sectional design and employed one or more of the following data collection methods: self-reporting, questionnaire-based surveys, and face-to-face structured interviews. Most of the studies collected information through a combination of interviews and questionnaire-based surveys [47,48,52,55,56,59,61,62,63,64,66,69]. In contrast, 10 studies relied solely on self-reporting [49,51,53,54,57,58,65,70,71,72]. Questionnaire-based surveys were the primary method in three studies [60,67,68]. One study [50] used a semi-structured questionnaire.

Sampling methodologies were described in 16 studies. Among these, five studies were conducted with simple random sampling [48,49,54,69,70], four [47,58,59,60] with convenience sampling, two [61,64] with multi-stage random sampling, two [67,68] with voluntary response sampling, two [56,63] purposive sampling, and one [57] with both non-probability and snowball sampling techniques.

### 3.5. Sociodemographic Characteristics of Consumers

Regarding sociodemographic characteristics, information on participants’ gender, age, education level, and economic status was extracted. Out of 26, 22 studies had both male and female participants, four had only female participants [47,51,52,65], and one did not specify any sex of the participants [61]. Among the 10 studies [48,49,53,56,59,62,63,66,68,69] the proportion of female participants was notably higher than the males. Seven studies focused on university students [49,53,54,58,60,65,72], two studies were on school children [70,71], four studies on street food consumers [57,62,67,68], five studies on meat consumers [50,56,59,64,66], and one study each on vegetable [48] and milk consumers [61]. The age of participants ranged between 7 and 78 years old. Four studies reported that a proportion of consumers had no formal education (ranging from 8% to 41%) [48,52,56,67], 13 studies reported consumers to have primary education (2–51%) [48,50,51,52,55,56,57,61,63,64,67,68,69], 12 studies reported secondary education (3–76%) [48,49,51,52,55,57,59,61,63,64,69,70], 13 studies reported tertiary education (17–70%) [48,49,52,55,56,57,59,63,64,66,67,68,69], and one study did not report the education level of the consumers [47].

### 3.6. Consumer Food Safety KAP Research in LLMICs

Most of the studies did not focus on a specific food category. However, six studies specifically examined prepared ready-to-eat foods and meat products. A detailed breakdown of studies by food category is presented in Table 4.

Regarding thematic focus, microbial contamination and foodborne pathogens were the most investigated topics (n = 22) (e.g., [47,57,67]). This was followed by studies addressing cross-contamination and hygiene practices (n = 10) (e.g., [52,69,70]). Few studies examined chemical contamination such as heavy metals and adulteration (n = 2) [48,61], food spoilage and quality issues (n = 2) [56,63], and zoonotic disease transmission (n = 1) [64].

#### 3.6.1. Food Safety Knowledge, Attitudes, and Practices (KAP)

Table 2 categorizes studies according to three consumer-related dimensions of food safety: knowledge, attitudes, and/or practices (KAP). Among the twenty-six studies, four [52,55,63,71] examined the full KAP triad. Ten studies [47,49,51,53,54,58,64,69,70,72] measured both food safety knowledge and practices, while three studies measured both knowledge and attitudes [63,67,68]. Nine studies [48,50,56,57,59,60,61,62,65] focused exclusively on food safety knowledge or awareness, and one study [66] examined food safety practices alone. Across studies that measured knowledge, attitudes, and self-reported practices, common topics included general food safety awareness, handwashing habits, household cross-contamination, food storage, food handlers’ hygiene as a source of illness, and the importance of appropriate cooking or refrigeration practices.

#### 3.6.2. Food Safety Knowledge

Twenty-five studies reported on consumer food safety knowledge and awareness, typically assessed using surveys with close-ended (true/false or multiple-choice) questions. No standardized measurement instrument was used across studies, and scoring methods varied considerably.

Overall, a substantial proportion of studies reported low or poor levels of knowledge [48,53,54,56,59,64,65,69,70,72]. Several studies reported good or satisfactory knowledge levels [49,50,51,52,57,58,60,63,68], while other studies described knowledge as average or mixed [47,55,61,62,67,71].

Numerous studies identified critical gaps in basic food safety understanding. For instance, one study [53] found that over half (53.6%) of Lebanese university students had low food safety knowledge, while another [54] reported a mean knowledge score of 41.8% among Bangladeshi students. Research by [64] observed limited awareness of safe cleaning and handling practices among Nigerian meat consumers. Similarly, this study [65] documented poor understanding of foodborne pathogens and safe cooking temperatures among Jordanian students. Studies focusing on school-aged children and youth populations [59,70,72] consistently revealed misconceptions related to food storage, cross-contamination, and hygiene.

Conversely, several studies reported relatively high levels of food safety knowledge. One study [58] found that more than half of Ghanaian students demonstrated good understanding of cross-contamination and handwashing practices. Two studies, [49,52], reported high knowledge levels of food safety among Jordanian university staff (90.6%) and Ethiopian mothers (75.9%), respectively. Similarly, studies [50,51] observed strong awareness of foodborne contamination risk among Ghanaian meat consumers and pregnant women. Furthermore, studies from Vietnam [63,68] documented good understanding of hand hygiene and separation of raw and cooked foods.

A smaller number of studies reported average or mixed findings. One study [55] found that consumers in Lao PDR had some general awareness but lacked the specific knowledge to effectively prevent foodborne diseases, particularly regarding microbial contamination. Studies [62,67] identified average knowledge levels among Vietnamese and Haitian consumers, with notable gaps in recognizing specific foodborne pathogens such as *Salmonella* spp. and *Staphylococcus* spp. A study conducted in Pakistan [47] reported fair knowledge among women. Another study [71] found that Sri Lankan schoolchildren performed well in areas such as personal hygiene and purchasing practices but showed moderate understanding of cross-contamination control.

Four studies specifically examined food safety awareness rather than broader knowledge constructs. One study [48] found poor awareness of heavy metal contamination among vegetable consumers in Nigeria, and another [56] reported limited awareness of meat safety issues in India. In contrast, there was [57] high awareness among Filipino street food consumers (82%), whereas Indian households [61] showed mixed awareness, with good understanding of milk safety but limited awareness of contamination sources and regulatory standards.

#### 3.6.3. Food Safety Attitudes

Seven studies assessed food safety attitudes, typically using direct survey-based approaches in which respondents were classified as having good or poor attitudes based on aggregated scores from factual and opinion-based questions (e.g., agreement with statements such as whether proper hand hygiene can prevent foodborne diseases). Overall, findings were mixed: one study reported predominantly poor attitudes [52], three reported average attitudes [62,67,71], one reported generally positive attitudes [68], and two showed mixed results [55,63].

For example, nearly half of Ethiopian mothers (49.6%) held negative food safety attitudes [52]. Conversely, 99% of Vietnamese street food consumers expressed strong positive attitudes towards basic hygiene practices, acknowledging that proper hand hygiene could prevent foodborne diseases [68]. However, attitudes were often internally inconsistent. In Haiti, while most respondents (82.5%) recognized the importance of hand hygiene, a substantial proportion (62.5%) incorrectly believed that well-cooked foods were always free of pathogens [67]. Similarly, in Vietnam, consumers prioritized freshness and cleanliness when choosing food outlets, yet 63% were unwilling to report safety violations to authorities [63].

#### 3.6.4. Food Safety Practices

Food safety practices were assessed in 15 studies, all of which relied on self-reported measures collected through multiple choice questionnaires. Most of these studies (n = 9) described practices as poor or unsatisfactory [51,52,53,54,55,64,66,69,72]. For example, in Ghana [51] more than half of pregnant women engaged in unsafe food handling behaviours, including unsafe thawing methods, inadequate hand hygiene, and wearing accessories while preparing food. Studies among university students in Lebanon and Bangladesh similarly reported low adherence to recommended food safety practices, particularly regarding cross-contamination and temperature control [53,54]. In Lao PDR, fewer than 10% of consumers consistently used separate cutting boards for raw and ready-to-eat foods [55], while in Nigeria, there was widespread unsafe meat-handling practices including inappropriate use of refrigeration and thermometers [64]. Other studies commonly reported high-risk behaviours, including improper storage, unsafe thawing, and poor cleaning of food contact surfaces [66,69,72].

In contrast, one study reported generally satisfactory food safety practices [71], and four studies reported good practices [47,49,58,70]. One additional study revealed a mixed picture [63]. In Ghana, 90.2% of students followed good practices, with a mean score of 36.2 achieving 80.4% of the maximum total food safety practice score [70]. Similarly, nearly half of women in Pakistan demonstrated good practices, particularly in food hygiene [47]. Of Jordanian university students and staff, 75.1% reported high-level of food safety practice, including consistent hand hygiene, washing of fruits and vegetables, and appropriate cleaning of utensils used for raw meat [49].

### 3.7. Factors Associated with Food Safety KAP

Twenty-two studies reported various sociodemographic and contextual factors associated with food safety knowledge, attitudes, and practices (Table 5). Age was a significant factor. For instance, food safety knowledge (*p* = 0.00008) and practices (*p* < 0.000017) were significantly associated with age among women in Bahawalpur City, Pakistan [47], while age was also positively associated with food safety knowledge in studies conducted in Ghana [50,60,70], Vietnam [62,68], Nigeria [64], and Jordan [49]. In Bangladesh, students older than 23 years old demonstrated significantly better food handling practices compared to younger students [54]. However, other studies such as [51,53,67] found no associations with age.

The relationship between educational attainment and food safety KAP was notably inconsistent across studies. In several studies, individuals with higher levels of education exhibited significantly better food safety knowledge and attitudes [57,61,62,63,65,68,72]. Yet this pattern did not hold for all the studies. Other studies [48,50,51,53,58,67] found no significant link between education and food safety outcomes. One study stood out [52]. Using odds ratios provided compelling evidence from Ethiopia where higher education levels were strongly associated with more positive food safety attitudes, highlighting that context and study design may play a critical role in these divergent findings.

Gender showed mixed associations across the studies. Some reported significant associations with food safety knowledge and practices [49,53,54,64,71], while others found no significant gender-based differences in knowledge or attitude [51,57,58,60,62,67,68,70].

Marital status was positively associated with food safety knowledge and practices in Jordan [49], and with knowledge scores in Vietnam [63]. One study [64] reported marital status was significantly associated (*p* < 0.05) with food safety knowledge but no significant association with food safety practices. Employment status was significantly associated with knowledge and practice in several contexts, including among pregnant women [51], general consumers [62], and university students [54,58]. In one study, children of working mothers scored better in food handling practices [53], while conversely another reported better scores in children of housewives [54].

Income level was also a significant predictor in some studies, particularly in Nigeria [48], Ethiopia [52], and Ghana [51,66], though other studies (e.g., [55,65]) found no significant associations. Notably, higher income and education levels were associated with better milk safety awareness among households in North India [61].

## 4. Discussion

This scoping review explored food safety KAP of urban consumers in low- and lower-middle-income countries (LLMICs). Searches identified a relatively small number of available studies (26) across 14 countries, which highlights the lack of information on this topic. This review focused on low- and lower-middle-income countries (LLMICs) to capture settings where rapid urbanization, the prominence of informal food markets, and constrained regulatory and enforcement capacities may disproportionately increase the risk of foodborne illnesses among consumers. Although LLMICs bear a high burden of foodborne disease [2] and are undergoing rapid transformations in urban food environments, consumer-focused evidence on food safety knowledge, attitudes, and practices remains relatively scarce compared with high-income countries [73,74]. Understanding consumers’ KAP is essential for improving food safety in urban LLMICs, where systemic limitations make individual behaviours the main defence against food-safety risks.

In the studies reviewed, measurement methods included self-reporting, questionnaire-based surveys, and face-to-face structured interviews. Studies mainly featured lower-middle-income countries with only two studies conducted in low-income countries. All the studies applied cross-sectional study designs. The analysis highlighted significant gaps in food safety KAP research among urban consumers. Only four studies [52,55,63,71] comprehensively examined all three components—knowledge, attitudes, and practices—which reveals a limited application of the KAP model in consumer food safety research in urban settings. Most studies reported knowledge and practices, while some studies assessed attitudes in combination with knowledge. Nine studies reported exclusively on knowledge and awareness, while only one study [66] measured practices in isolation. Studies that identified poor food safety knowledge among respondents also reported correspondingly poor food safety practices. However, seven studies showed that respondents’ knowledge was better than their self-reported practices [49,51,52,53,54,58,64]. Studies that explored multiple dimensions of food safety including knowledge, attitudes, and practices mainly focused on specific themes such as hand hygiene, cross-contamination prevention, food storage practices, and the role of food handlers, indicating the significance of both individual and systemic determinants influencing food safety behaviour.

A consistent, critical, finding across this review was the recurring discrepancy between high levels of food safety knowledge and poor actual practices among urban consumers in LLMICs. While several studies identified that poor knowledge directly corresponded to poor practices, a significant number of studies [46,48,49,50,51,55,61] revealed that consumers often possessed adequate theoretical knowledge regarding food safety—such as the need for handwashing or proper storage temperatures—but failed to apply this knowledge in their daily routine. This observed gap between knowledge (and, in some cases, attitudes) and practices may partly reflect reporting bias, including social desirability bias, whereby respondents underreport undesirable behaviours and overreport socially acceptable practices [75]. Other KAP studies identified similar gaps. For example, reviews conducted in Nigeria and Ethiopia found that while the majority of included studies reported “good” levels of food safety knowledge among consumers, most also documented persistently “poor” food safety practices [6,33].

These findings suggest that interventions focused solely on improving food safety knowledge are unlikely to be sufficient. Rather, the persistence of the knowledge–practice gap indicates that structural, cultural, and environmental constraints play a critical role in shaping consumer behaviour. Such constraints may include limited access to safe water, inadequate household or community infrastructure in urban settings, time constraints related to urban livelihoods, and economic barriers that restrict access to appropriate food storage or preparation equipment. There is a clear need to move beyond information-based approaches and to address the broader systemic and environmental determinants that influence the adoption of safe food handling practices.

Food safety knowledge presented mixed findings across the included studies. While some studies (e.g., [49,51,60]) reported good or satisfactory levels of knowledge, ten studies (e.g., [53,65]) found low or poor levels and substantial knowledge or awareness gaps, while six studies [47,55,61,62,67,71] showed average or mixed findings. Inconsistencies in food safety knowledge levels across countries may be the result of factors such as cultural practices and beliefs, education levels, and varying effectiveness and accessibility of public health messaging. Regarding food safety awareness, two studies [48,56] focusing on leafy vegetables and meat food quality and safety reported poor levels of awareness. On the other hand, only one study on street food consumers [57] found good levels, while another study on milk-consuming households showed a mixed outcome [61]. Common knowledge issues were poor knowledge of pathogens, for example, unawareness of microbial causes of foodborne disease (e.g., [55,62,65,67]), lack of knowledge of how to prevent transfer from raw to ready-to-eat food (e.g., [55,72]), and lack of knowledge on refrigeration, thawing, and safe cooking temperatures (e.g., [65,70]).

In contrast, few studies included assessment of consumer attitudes toward food safety, which also reported inconsistent findings making it difficult to respond to that aspect of the research question. While three studies [62,67,71] found adequate and satisfactory attitudes, one [52] identified poor attitudes, and other studies [55,63] revealed mixed results. Common issues were incorrect beliefs about thawing, contamination in cooked foods, egg washing [67,68], poor personal responsibility towards food safety [52], and low engagement in food safety enforcement/reporting [63]. None of the studies explicitly addressed cultural beliefs. The prevalence of poor safety practices is a significant issue confirmed by this review.

Among the 15 studies (e.g., [53,54,58]) which assessed practices, the majority found that practices were poor among consumers, while only limited studies reported good [47,58,70], satisfactory [71], or very good practices [49]. Cross-contamination, poor hand hygiene, and unsafe thawing/temperature control were the most reported issues. They may occur for many reasons due to lack of knowledge/awareness of best practices [54] and limited access to food thermometers [58]. These findings highlight potential areas for interventions to improve food hygiene practices among urban consumers in LLMICs.

Several socio-demographic and contextual factors were associated with food safety KAP among urban consumers in LLMICs. Findings may provide valuable insights regarding social and structural drivers that impact consumer behaviour. These can be used to develop and implement specific, focused interventions. Age was significantly associated with at least one component of food safety KAP across diverse populations (e.g., [47,49,50,60,68,70]). Only one study found that younger consumers were more likely to demonstrate better food safety knowledge and attitudes [68].

Educational attainment was a strong determinant of at least one component of food safety KAP. Several studies showed that higher education levels were significantly associated with better KAP scores across various settings and populations (e.g., [52,62,64,68,72]). The importance of food-related education (e.g., nutrition, food science) and food safety training [65,68] was highlighted as a significant factor in improving both knowledge and attitudes.

Marital status and employment status also showed significant associations in a few studies (e.g., [49,51,63]). Employed individuals and those who were married were inclined to have better knowledge and practice scores, potentially because of more food preparation responsibilities within households, or better access to resources and information.

By contrast, gender showed inconsistent associations with food safety KAP. While some studies reported significant associations with at least one component of KAP (e.g., [54,58]), others found no significant gender differences (e.g., [62,67,68,70]). This indicates that gender differences may be context-specific and are shaped by other factors like education levels or family roles.

Income levels also emerged as a significant factor. Evidence from studies, for example, conducted in Ethiopia [52], India [61], and Ghana [66] showed that higher household income was positively associated with at least one component of food safety KAP. This may be potentially due to better access to clean, safe food environments as well as health information.

### 4.1. Research Gaps

We identified several gaps in this scoping review that warrant further investigation. First, although studies were conducted across multiple LLMIC regions, geographical representation was uneven, with fewer studies from sub-Saharan Africa and Latin America compared with South Asia and Southeast Asia. Evidence from low-income countries was particularly limited, restricting understanding of food safety KAP among urban consumers in these settings. Of the 26 included studies, 92% were conducted in lower-middle-income countries, while only two studies were from low-income countries (Ethiopia and Sudan), and none used multi-country or comparative designs. This imbalance limits the ability to assess regional or income-level patterns in food safety KAP. Although variation was observed across individual country contexts, such differences cannot be confidently attributed to region or income level due to limited representation and heterogeneity in study designs and measurement tools. Overall, the concentration of evidence in lower-middle-income countries highlights a critical gap and underscores the need for future research in low-income and under-represented regions, ideally using standardized and comparative approaches to enable more robust assessment of contextual patterns in food safety KAP.

Second, all included studies relied on cross-sectional close-ended surveys to assess KAP. Evidence from consumer food safety research indicates that surveys are the most commonly used method in this field [27]. While surveys are efficient for rapidly collecting data and providing a snapshot of a population, they present several limitations for assessing food safety KAP. In particular, reliance on self-reported practices makes findings susceptible to social desirability and recall bias [76,77], potentially resulting in an overly optimistic assessment of food safety knowledge, and behaviours compared with alternative approaches [27]. Moreover, cross-sectional designs limit causal inference, making it difficult to understand how food safety KAP evolves over time. Longitudinal studies would instead provide a deeper understanding of food safety behaviours and the factors influencing their change. Furthermore, studies varied widely in scoring systems, domains assessed, and validation status, with several studies examining only one or two KAP components and classifying them dichotomously as “good” or “poor.” The lack of standardized definitions and measurement approaches for key constructs—particularly attitudes and practices—hinders comparability across countries and studies and constrains the potential for synthesis, including meta-analysis. Developing standardized indicators, improving questionnaire design, and validating measurement tools are therefore critical for advancing consumer food safety KAP research. Also, integrating observational methods with qualitative approaches, such as in-depth interviews, would allow researchers to ask follow-up questions and probe certain issues in greater depth.

Third, the lack of consistent data on attitudes is a significant research gap since food safety attitudes play a crucial role in influencing food safety behaviours and often act as a mediator between knowledge and practices. Studies which examined food safety attitudes often interpreted attitudes from answers to close-ended questions instead of analysing the topic in detail, for example via unstructured interviews, which could be more useful for nuanced topics. Moreover, definitions of attitudes and how they were distinct from knowledge or practices were often unclear. Inconsistencies and overlapping definitions are also prevalent in food safety research among high-income countries [78]. These are key gaps since an in-depth understanding of consumers’ beliefs, motivations, and other aspects of attitudes may be required for the design of effective food safety strategies and interventions for behaviour change [79,80,81]. Inconsistencies and overlapping definitions are also prevalent in food safety research among high-income countries [78]. These are key gaps since an in-depth understanding of consumers’ beliefs, motivations, and other aspects of attitudes may be required for the design of effective food safety strategies and interventions for behaviour change [79,80,81].

While socio-demographic factors such as education and income were frequently cited, there was a notable lack of attention to cultural beliefs, levels of urbanization, and food market structures. This omission is important, as food safety practices in LLMICs are often shaped by traditional food cultures and by the physical and regulatory conditions of informal food markets, including “wet” markets. The predominant focus on individual-level characteristics in the existing literature, therefore, risks overlooking the systemic, environmental, and institutional drivers that constrain or enable safe food handling behaviours. There is therefore a need for future research to incorporate cultural frameworks (e.g., using ethnographic methods, belief surveys, or participatory research). Such insights may help educators to understand what influences a consumer to invest effort in implementing practices or otherwise what barriers to implementation of improved practices may exist. Nevertheless, this review reveals that no research has been conducted on this issue among urban consumers in LLMICs, highlighting an important priority for future research.

Future studies that aim to assess food safety through observed consumer behaviours and/or knowledge surveys should also investigate all aspects of consumer attitudes regarding food safety. This qualitative and observational research method will likely require more time and resources compared to close-ended surveys. Furthermore, observational studies may be challenging to conduct in certain cultures since some respondents may not consent to be observed, and close supervision is crucial for ensuring responsible and ethical research practices. Moreover, the high proportion of studies assessed as low quality reflects limitations in the existing evidence base and highlights the need for more robustly designed consumer food safety KAP studies in urban LLMIC settings.

Fourth, another clear research gap is the lack of experimental methods to understand choices. None of the included studies used experimental or behavioral economics designs (discrete choice experiments, behavioral simulations, or randomized trials) for understanding consumer food safety choices. Such methods have been successfully applied in many LMICs [22,82].

### 4.2. Implications for Policy

The evidence synthesized in this review highlights several implications for the design of food safety interventions. While studies captured components of the KAP (knowledge, attitudes, practices) framework, few applied the model in its entirety providing an incomplete picture of behaviour change. According to the KAP model, knowledge is expected to influence attitudes, which in turn drives practices. However, this fragmented application across the reviewed studies has limited our understanding of how food safety knowledge influences attitude and ultimately translates into actual safe food handling behaviours. It is therefore essential for policy makers to design interventions that integrate KAP components. Such interventions should go beyond increasing knowledge to also enhance motivation, for example, by providing behavioural nudges and incentives [83] for influencing practices. Crucially, this includes the modernization of urban street food market regulations; rather than adopting purely punitive measures, policies should focus on “supportive regulation” that provides vendors with the infrastructure necessary for compliance, such as access to clean water and waste disposal. This is different from what was recommended in most of the reviewed studies, which primarily emphasized education and raising awareness, even when the studies themselves did not confirm a direct link between a lack of knowledge and poor practices. However, this suggestion is consistent with previous systematic reviews focused on high-income countries [78], which have concluded that strategies beyond just education are needed to effectively change food safety behaviours. Education alone may not be sufficient to ensure food safety practices. Therefore, future consumer education curriculum design must shift from theoretical knowledge to action-oriented skills. Curricula should be designed to address “critical control points” at the household level, using visual-heavy and skill-based learning modules that are accessible to populations with varying literacy levels. Moreover, understanding underlying attitudes and beliefs is crucial for designing effective interventions that address motivations.

Overall, some of the included studies reported positive associations between educational attainment, income levels, and food safety KAP. Given this, programs and interventions may be targeted based on socio-demographic profiles. Our review underscores the need for targeted interventions tailored to particular groups and contexts such as low-income, less-educated urban consumers. These interventions must be culturally sensitive and context specific. For instance, in urban settings with limited formal education, using local languages and combining traditional food safety practices with modern guidelines could improve effectiveness of programs targeted towards consumers.

On the other hand, some studies reported inconsistent associations between gender and food safety KAP. It may be that gender-related differences are context-specific and mediated by other variables such as education or household roles. Typically, women are mostly food handlers and, therefore, it is necessary to empower them in safe food handling practices [84]. Addressing these gendered dimensions of food safety would entail providing training programs on food handling practices, food hygiene, and safety [85]. These programs would focus on raising women’s knowledge and skills in food safety, particularly for those who are primary caregivers and food handlers.

### 4.3. Strengths and Limitations

This is the first scoping review focusing exclusively on the food safety KAP of urban consumers in LLMIC. The strengths of this review include the use of the PRISMA-ScR guidelines to ensure a robust and replicable process, the use of seven electronic databases to capture the breadth and depth of peer-reviewed publications, and the quality assessment of all the studies. Furthermore, the review identifies key research gaps and provides valuable policy insights. We also, however, acknowledge several limitations. First, to ensure the viability of this scoping review, our emphasis was on the established food safety terminology. Although we relied on prior reviews to generate our syntax of search terms, we acknowledge that there is a wealth of relevant research from wide-ranging disciplines that may apply alternate nomenclature such as “food quality” or “food spoilage”. Second, this review focused on a broad construct of “knowledge, attitudes, and practices” and incorporated a variety of different populations. The ability to synthesize disparate literature is a key strength of scoping reviews. However, by including such a broad range of studies, it also limits the scope to undertake a fine-grained analysis that other systematic review styles with a narrower focus provide. Third, this review only focused on LLMICs, which excluded research in upper-middle-income countries. However, this narrower scope is also a strength, as it allows the analysis to be more targeted and specific. Fourth, we restricted to studies published in the English language which may have excluded key studies written in other local languages. Fifth, by restricting our search to only peer-reviewed journal articles, it is likely that we may have excluded any potentially relevant grey-literature publications. Furthermore, by exclusively relying on studies that were available electronically, it is possible that we may have missed studies for which full-text versions were not available online. Sixth, the high degree of methodological heterogeneity across the 26 included studies—including diverse sampling techniques, non-standardized KAP survey instruments—precludes a formal meta-analysis or a direct statistical comparison of results. In alignment with PRISMA-ScR guidelines, our objective was to map the breadth of evidence. Consequently, the synthesis remains descriptive to avoid over-generalizing findings from distinct urban contexts in LLMICs where local food environments significantly differ.

## 5. Conclusions

This scoping review synthesized evidence from 26 studies on food safety knowledge, awareness, attitudes, and practices (KAP) among urban consumers in 14 LLMICs, highlighting significant gaps. The main topics of study were knowledge and self-reported practices; attitudes were less commonly studied. Both food safety knowledge and attitudes reported inconsistent findings while practices were shown to be poor among consumers. In general, studies indicated a lack of translation of knowledge and attitudes to practice. Furthermore, the reviewed studies had several methodological gaps and weaknesses such as unclear sampling strategy or sample representativeness, missing information on response rates and potential sources of bias, and lack of standardized KAP assessment tools. The paucity of evidence from high-quality studies is an important concern when drawing inference about the overall food safety KAP among consumers in LLMICs. There is an urgent need to conduct high-quality studies with improved study designs, methods, and metrics with less reliance on close-ended survey questions and self-reported data. Integrating more direct observations and in-depth qualitative methods will better capture consumer food safety KAP. Improving the quality of research and filling the much-needed knowledge gap will be crucial in influencing a positive change in food safety behaviour. It should be made a priority as foodborne disease is likely to become increasingly problematic as urbanization and economic development continue in LLMICs.

## Figures and Tables

**Table 1:** PCC model of the scoping review.

Population (P)	“Consumers”: individuals purchasing and/or acquiring food for themselves or their families
Concept (C)	Knowledge, attitude, and/or practices (KAP) related to food safety Knowledge refers to individuals’ understanding of personal hygiene practices, methods for preventing cross-contamination, and acknowledging the causes and symptoms of foodborne diseases Attitudes relate to individuals’ beliefs, opinions, and perceptions concerning practices such as handwashing, cross-contamination prevention, and safe food handling and storage Practices pertain to observable behaviours such as personal hygiene routines, compliance with handwashing protocols, and appropriate food handling and storage practices
Context (C)	Urban settings in LLMICs

**Table 2:** Key characteristics of all included studies (n = 26).

Study	Study Setting and Location	Data Collection	Study Design and Method	Respondents and Sample Size	Education Status	Economic Status	Aspects Assessed (KAP)
[47]	Bahawalpur City, Pakistan	April–September 2021	Cross-sectional; Survey (WHO questionnaire), face to face interview	Women (n = 193). Age groups 15–19 (n = 4), 20–29 (n = 19), 30–39 (n = 39) and 40–49 (n = 77) years	Not reported	Not reported	Knowledge and practices
[48]	Kano, Nigeria	November–December 2023	Cross-sectional; Survey, face to face interview	86 leafy vegetable habitual consumers (n = 64 male & n = 24 female). 74.42% male, 22.58% female. Age groups: <20 (4.6%), 20–30 years (37.2%), 31–40 (34.9%),41–50 (15.1%), 51–60 (7%), >60 (1.2%).	75.6%—secondary education. 18.6%—non-formal education, 5.8%—primary school, 41.9%—secondary school, and 33.7%—tertiary institutions	83.72% are self-employed	Awareness
[49]	Zarqa University, Jordan	December 2022–January 2023	Cross-sectional; Survey, Self-reported questionnaire	Students and staff members (n = 406); age: 18–50 years. Males (n = 153) and females (n = 253). 62.3% female, 37.7% male. Student respondents (n = 219; 53.9%).	B.Sc. Students (n = 219) (53.9%); B.Sc. Level (n = 96) (23.6%); postgraduates (n = 55) (13.5%) secondary education (n = 36) (8.9%)	Not reported	Knowledge and practices
[50]	Tema Metropolis, Ghana	Not reported	Cross-sectional; Survey, Semi-structured questionnaire	Meat consumers (n = 384). 21–40 years (54%). Males (56%).	51% with basic education.	Not reported	Knowledge
[51]	Assin Foso, St. Francis Xavier Hospital, Ghana	November 2017–December 2017	Cross-sectional; Survey, Self-reported questionnaire	Pregnant women (n = 170) attending ANC services. <18 years = 4.12%, 18–24 years = 21.18%, 25–34 years = 59.41%, 35+ years = 15.29%.	Primary education—7.65%, Junior high—52.94%	Full-time employees—52.35%	Knowledge and practices
[52]	Debarq town, Amhara region, Northwest Ethiopia	2019	Cross-sectional; Questionnaire, face to face interview	Mothers of under-5 children (n = 423). Mean age—39.844 ± 11.02 (SD).	Diploma and above—16.8%. Primary education—2.7%. Secondary education—19.9%. 40.7% did not read and write.	<800 birr= 26.2%, 800–1100 = 24.3%, 1101–2145 = 24.6%, >2145 = 24.8%	Knowledge, attitude and practice
[53]	Beirut and Byblos of the Lebanese American University (LAU), Lebanon	November–May 2013	Cross-sectional; Survey, Self-reported questionnaire	Lebanese university students (n = 1172). Mean age—20.0 (SD −1.7). 59% females and 41% males.	4% freshmen, 39% sophomores, 30% juniors, 26% seniors.	Not reported	Knowledge and practices
[54]	Patuakhali Science and Technology University (PSTU), Jashore University of Science and Technology (JUST), Hajee Mohammad Danesh Science and Technology University (HSTU), and Islamic University (IU), Bangladesh	January–March 2022	Cross-sectional; Survey, Self-reported questionnaire	University students (n = 1534). Male 50.6%, female 49.4%. Mean age = 22.09 (SD −1.78).	B.Sc. 1st year—35.7%, B.Sc. 2 nd year—24.3%, B.Sc. 3rd year—17.5%, B.Sc. 4th year—14.4%, Masters’—8.1%	Monthly income: Up to 15,000 BDT = 27.3%, 16,000 to 30,000 = 45.4%, Above 30,000 = 27.3%	Knowledge and practices
[55]	Seven districts of Vientiane Capital, Lao People’s Democratic Republic (PDR)	March–April 2022	Cross-sectional; Survey, face to face interview	n = 500. 47.8% women, 41.6% men. Age groups: 14–25 years (35.8%), 26–35 years (31.8%), 36–55 years (24.8%), 56–78 years (7.6%).	Less than 5 years primary school—3.8%. Primary school—6.6%. Junior high school—12.0%. Secondary school—32.2%. Vocational technical school—15.8%. Bachelor’s degree—27%. Master’s degree—2.6%	Income level: Very low—3.3%. Low—8.2%. Average—25.6%. Above the average—23.3%. Significantly above the average—39.6%.	Knowledge, attitude and practices
[56]	Bengaluru District of Karnataka, India	Not reported	Cross-sectional; Survey, face to face interview	Males (n = 194) and females (n = 66). Age groups: 16–30 years (28.1%), 30–44 years (55%), 44–58 years (16.9%).	Illiterate—10.4%, Primary—16.9%, Undergraduate—46.1%, Postgraduate—26.6%.	Not reported	Awareness
[57]	Manila City, Philippines	Not reported	Cross-sectional; Survey, Self-reported questionnaire	Respondents (n = 384): male (n = 189; 49.2%) and female (n = 195; 50.8%).	College undergraduate (n = 224; 58.3%), College graduate (n = 92; 24%), Junior high school (n = 10; 2.6%), Senior high school (n = 55; 14.3%), Elementary (n = 3; 0.8%).	Not reported	Awareness
[58]	University for Development Studies and Tamale Technical University, Northern Region of Ghana	February 2022–May 2022	Cross-sectional; Survey (WHO questionnaire), Self-reported questionnaire	Students (n = 397). Males (n = 202 (50.9%)), females (n = 195 (49.1%)). Age group: 22 to 25 years—51.9%, 30 years and above—5.5%.	University students	Not reported	Knowledge and practices
[59]	Cau Giay district, Hanoi and Da Bac district, Hoa Binh province, Northern Vietnam	November–December 2019	Cross-sectional; Survey, questionnaire, face to face interview	Participants (n = 225) (76 from rural traditional markets, 76 from urban traditional markets, 73 from urban modern markets). Age groups: 18–25 (modern urban-21, traditional urban-3), 26–35 (modern urban-30, traditional urban-12), 36–45 (modern urban-11, traditional urban-11), 46–55 (modern urban- 3, traditional urban-20), >56 (modern urban-6, traditional urban-30). 81.8% female.	Secondary school: modern urban—4.1%, traditional urban—30.3%; High school: modern urban—35.6%, traditional urban—46.1%; Colleges/university or higher: modern urban—60.3%, traditional urban—15.8%	Not reported	Knowledge
[60]	Ho Technical University (HTU), University of Allied and Health Sciences (UHAS), and Evangelical Presbyterian University College (EPU), Ghana	August 2021–February 2022	Cross-sectional; Survey, questionnaire	University students (n = 803). Males (n = 420 (52.3%)), female (n = 383 (47.7%)). Age groups: less than 20 (15.2%), 20–25 (52.8%), 26–30 (30.3%), and above 30 (1.7%).	HND—41.5%, Bachelor of Science—46.1%, B-Tech—11.5%	Not reported	Knowledge and awareness
[61]	North India (New Delhi, Ludhiana, Karnal)	February–April 2022	Cross-sectional; Survey, face to face interview	Milk-consuming households (n = 270). Average age of the household head—44.69 years.	82% of household heads have at least high school education, 14%—middle level education, and 4%—primary education	Average annual household income—Rs. 604,000. Karnal (Rs. 678,993), New Delhi (Rs. 589,467) and Ludhiana (Rs. 543,600).	Awareness
[62]	Can Tho City (five districts—Ninh Kieu, Cai Rang, O Mon, Phong Dien and Binh Thuy), Vietnam	November 2016–October 2018	Cross sectional; Survey, questionnaire, face to face interview	Street food consumers (n = 838). Females (n = 535; 63.8%) and males (n = 303; 36.2%). Mean age 26.4 ± 10.35 years.	84% (n = 705) have at least high school level of education	66% (n = 555) of consumer-respondents have monthly income of at most 3 million VND; 27% (n = 225) are earning between 3–8 million VND; 7% (n = 58) are earning at least 8 million VND.	Knowledge, attitude and practice
[63]	Hanoi, Vietnam	September–October 2015	Cross sectional; Survey, face to face interview	1760 participants recruited, with 1740 qualified for analysis. Male (n = 663); female (n = 1077). 61.9% female. Mean age: 34.6 (SD = 12.9).	55.9% of respondents have more than a high school education (college, university). 31.1%—high school, 10.5%—secondary school, 2%—primary school	59% of respondents have some type of work (i.e., office worker, worker, employed in other jobs) while the rest are students (17.8%), retired (7.3%), housewife (13.4%), or unemployed (2.1%)	Knowledge, attitude and practices
[64]	Ilorin City, Northcentral Nigeria	November 2019–January 2020	Cross sectional; Survey, questionnaire, face to face interview	Consumers (n = 869); Male (n = 392, 45.1%), female (n = 477, 54.9%). Female within the age range of 19–25 years (54.2%).	Primary education—2.5%, Secondary education—14.4%, Undergraduate—50.5%, Graduate—22.2%, Postgraduate—10.4%	24% (n = 206)—private business owners, 23% (n = 198)—civil servants; 54% (n = 465)—students	Knowledge and practices
[65]	Irbid City, Jordan	February–November 2009	Cross-sectional; Questionnaire, Self-reported questionnaire	Female college students (n = 867). Mean age of 20.07 (SD 1.81).	17.5% freshmen, 21.9% sophomores, 23.8% juniors, and 36.8% seniors	67.5% had family income level 600JD ($845) or more per month	Knowledge
[66]	Greater Accra Region of Ghana	Not reported	Cross sectional; Survey, questionnaire, face to face interview	471 recruited consumers, with 470 qualified for analysis (male, n = 158; female, n = 312). 33.6% male, 66.4% female. Age groups: 18–35 (66.4%), 36–50 (21.3%), >50 (12.3%).	Tertiary education—330 (70.2%)	A combined majority (66.9%) of respondents reported monthly incomes above the urban median income in Ghana (USD $68). Unemployed—6.4%	Practices
[67]	Port-au-Prince City, Haiti	July 2012–September 2012	Cross-sectional; Questionnaire, filled in either by the participants themselves or by the researcher for illiterate participants.	Consumers (n = 160). Male (n = 100, 62.5%), female (n = 60, 37.5%). Mean age of the participants was 29.6 ± 11.3 years. Age: 15–25 (46.2%), 26–35 (30.6%), 36–45 (14.4%), 46–55 (3.8%), 56–60 (3.1%), >60 (1.9%).	Illiterate—8.2%, primary school—15.6%, high school—43.1%, university—33.1%	Not reported	Knowledge and attitudes
[68]	Ho Chi Minh city (Binh Thanh (BT), Thu Duc (TD), district 3 (D3) and district 8 (D8)), Vietnam	July–August 2014	Cross-sectional; Questionnaire, filled in either by the participants themselves or by the researcher for illiterate participants.	Consumers (n = 120). Female (n = 72, 60%), male (n = 48, 40%). Age groups: 18–25: 66 (55%), 26–35: 23 (19.2%), 36–45: 19 (15.8%), 46–55: 6 (5%), 56–50: 6(5%).	Primary school 10 (8.3%), High school 38 (31.7%), University 72 (60.0%)	Not reported	Knowledge, attitudes and practices
[69]	Khartoum City, Sudan	March–May 2008	Cross sectional; Questionnaire, face to face interview	Consumers (n = 50). Male (n = 1, 2%); 49 female (n = 49, 98%). 46% aged between 18–34 years.	Primary education—18%, general secondary—18%, high secondary—44%, graduate—20%.	Not reported	Knowledge and practices
[70]	Ga West Municipality, Ghana	January–August 2019	Cross-sectional; Survey, Self-reported questionnaire	Upper primary and junior high school students (n = 1343). Male (n = 649, 51.7%); 694 female (n = 694, 48.3%). Mean age 13 years (SD- 1.9) ranging from 7–21 years.	Upper primary and junior high school students	Not reported	Knowledge and practices
[71]	Colombo, Sri Lanka	September–October 2020	Cross-sectional; Survey, self-reported questionnaire	School children (n = 380). Male—56.32%, female—43.68%. 34.74% (14 years), 31.32% (15 years), and 33.95% (16 years).	School children	Not reported	Knowledge, attitude and practices
[72]	University of Agriculture Peshawar, Pakistan	September–December 2014	Cross-sectional; Survey, Self-reported questionnaire	University students (n = 311) from food and non-food related departments aged 21–26 years old. Male (n = 165, 53.5%), female (n = 146; 46.95%). 42.76% aged 23–24, 35.04% aged 21–22, 22.18% aged 25–26.	University students	Not reported	Knowledge and practices

**Table 4:** Food groups analysed in the included studies.

Food	N	Reference
Leafy vegetables	1	[48]
Meat	6	[50,56,59,64,66,69]
Milk	1	[61]
Prepared ready-to-eat foods ^1^	6	[57,60,62,63,67,68]
Not specified/No food focus	12	[47,49,51,52,53,54,55,58,65,70,71,72]

^1^ refers to street food, takeaway meals, or food consumed without further cooking.

**Table 5:** Influence of sociodemographic and economic factors on food safety KAP.

Significant Variables	References for with Association on Knowledge	References for with Association on Attitude	References for with Association on Practices
Gender	[49,54,64,71,72]		[49,53,54,64,71,72]
Age	[47,49,50,58,60,62,64,68,70,72]	[62,68]	[47,49,54,70]
Education	[50,61,62,63,64,68]	[52,57,62,67,68]	[51,64,66,71,72]
Marital status	[49,63,64]		[49]
Number of children	[51]		
Location	[68]	[62,68]	
Residence area	[54]		[53]
Residential status	[53,54]		
Major of study/department of study/field of study	[53,54,58,60,65,72]		[53,54]
Year of study/level of study/class level	[58,70]		[54,70]
Academic performance (Science marks, Rank in class)	[71]	[71]	[71]
Education of parents	[71]	[71]	
Educational institution	[60]		
Tribe	[60]		
Income	[61,63]	[52,63]	[66]
Occupation			[65,66]
Employment	[51,62,63]		[51]
Maternal employment	[72]		[53,54]

Notes: ref. [63] under Employment category—the variable tested in the study was ‘blue collar worker’; belongs to poorest income quantiles, tended to have lower knowledge scores; ref. [63] under Income—the variable used was ‘Household belonging to poorest income quantiles’ which has negative association with knowledge about practices on raw and cooked foods; Samapundo et al. (2016) [68] under Education—positive influence on consumers; ref. [71] under Gender—the variable used was ‘Male’; higher food safety knowledge scores for males compared to females; however, food safety practice scores tend to decrease with Males; ref. [71] under ‘Science’ marks and rank in class—food safety scores tend to increase with these two variables; ref. [71] under Education of parents—average food safety score of school children with parents having lower level of education tend to decrease compared to parents who are diploma holders.

## Data Availability

No new data were created or analysed in this study.

## Supplementary Materials

The following supporting information can be downloaded at: https://www.mdpi.com/article/10.3390/foods15081381/s1., References [17,86,87,88,89,90,91,92,93,94,95,96,97,98,99,100,101,102,103,104,105,106,107,108,109,110,111,112,113,114,115,116,117,118,119,120,121,122] are cited in the Supplementary Materials.
