# Supplementary material for: Food Safety Knowledge, Attitude, and Practices (KAP) of Urban Consumers in Low-Income and Lower-Middle-Income Countries (LLMICs): A Scoping Review
Source: Foods. 2026 Apr 16;15(8):1381. doi: 10.3390/foods15081381 (PMC13114813; doi:10.3390/foods15081381)
Supplement: Supplementary file 1 [file foods-15-01381-s001.zip › foods-4127423-supplementary.pdf]

Table S1. Search strategy –PubMed (adult[MeSH] OR adult* OR "general population" OR adolescent[MeSH] OR adolescent* OR consumer*) AND ("food safety" OR "food borne" OR foodborne OR "food handling" OR "food preparation" OR "food poisoning" OR "food hygiene" OR "safe food" OR "food contamination" OR "food packaging" OR "food storage" OR "food scare*" OR "food sanitation" OR "food quality" OR "food adulteration" OR "food inspection") AND ("knowledge" OR "awareness" OR "belief*" OR "attitude*" OR "opinion*" or Pract* or Priori* or Expect* OR "Knowledge, Attitude, and Practices") AND (Afghanistan OR Albania OR Algeria OR Angola OR Argentina OR Armenia OR Azerbaijan OR Bangladesh OR Belarus OR Belize OR Benin OR Bhutan OR Bolivia OR Bosnia OR Herzegovina OR Botswana OR Brazil OR Bulgaria OR Burkina Faso OR Burundi OR "Cabo Verde" OR "Cape Verde" OR Cameroon OR Cameron OR "Central African Republic" OR Chad OR-China OR Columbia OR Colombia OR Comoros OR Comoro OR Congo OR "Costa Rica" OR "Ivory Coast" OR "Côte d'Ivoire" OR Cuba OR Djibouti OR Dominica OR "Dominican Republic" OR Ecuador OR Egypt OR "El Salvador" OR Eritrea OR Eswatini OR Ethiopia ORFiji OR Gabon OR Gambia OR Georgia OR Grenada OR Ghana OR Guatemala OR Guinea OR Guyana OR Guyane OR Haiti OR Honduras ORIndonesia OR India OR Iran OR Iraq OR Jamaica OR Jordan OR Kazakh* OR Kenya OR Kiribati OR Korea OR Kosovo OR Kyrgyz* OR Kirghizia OR Lao OR Laos OR Lebanon OR Lesotho OR Liberia OR Libya OR Madagascar OR Malawi OR Malaysia OR Mali OR Maldives OR "Marshall Islands" OR Mauritania OR Mexico OR Micronesia OR Moldova OR Mongolia OR Montenegro OR Morocco OR Mozambique OR Myanmar OR Burma OR Namibia OR Nauru OR Nepal OR Nicaragua OR Niger OR Nigeria OR Macedonia OR Pakistan OR Paraguay OR Peru OR Philippines OR Russia OR "Russian Federation" OR Rwanda OR Ruanda OR Samoa OR "Sao Tome" OR Senegal OR Serbia OR "Sierra Leone" OR "Solomon Islands" OR Somalia OR "South Africa" OR Sudan OR "Sri Lanka" OR "St. Lucia" OR "Saint Lucia" OR "St. Vincent" OR Grenadines OR Suriname OR Swaziland OR Syria OR Tajikistan OR Tadjikistan OR Tadzhikistan OR Thailand OR Tanzania OR "Timor-Leste" OR "East Timor" OR Togo OR Tonga OR Tunisia OR Turkey OR Turkmenistan OR Tuvalu OR Uganda OR Ukraine OR Uzbekistan OR Vanuatu OR Venezuela OR Vietnam OR "West Bank" OR Gaza OR Yemen OR Zambia OR Zimbabwe OR "developing countr*" OR "developing world" OR "low income countr*" OR "middle income countr*" OR "low middle income countr*" OR lmic OR lmics OR "third world" OR "emerging nation*" OR "emerging countr*" OR "global south" OR "under developed nation*").

Table S2. List of studies excluded after full text screening.

**List of studies**<br>**Study**	**Reference**	**Reason for exclusion**
Consumer food safety awareness and knowledge in Nigeria	[86]	No results for urban LMIC
Knowledge and awareness about food safety foodborne and microbial hazards: A cross-sectional study among Bangladeshi consumers of street-vended foods	[87]	No results for urban LMIC
Assessment of the knowledge, attitude and practice of childbearing mothers in the geographically coastal region of Bangladesh for cleanliness and food safety of food security	[88]	No results for urban LMIC

Milk handling practices and consumption behaviour among Borana pastoralists in southern Ethiopia	[89]	No results for urban LMIC
Food safety knowledge, attitudes, practices of traditional fish handlers and consumers in Ghana	[90]	Full text paper unavailable
Awareness regarding meat hygiene practices followed in Srinagar city of Jammu and Kashmir, India	[91]	Full text paper unavailable
Food Safety Knowledge, Attitude, and Practice of College Students, Ethiopia, 2019: A Cross-Sectional Study	[92]	Not peer-reviewed
Raw meat consumption and food safety challenges: a survey of knowledge, attitudes and practices of consumers in Lebanon	[93]	No results for urban LMIC
Focus group studies on food safety knowledge, perception and practices of school-going adolescent girls in South India	[94]	No results for urban LMIC
Awareness and practices among dairy producers and consumers in Sri Lanka	[95]	Not peer-reviewed
Socio-demographic determinants of knowledge, attitude and practices towards food safety among Lebanese population during the economic crisis: a cross-sectional study	[96]	No results for urban LMIC
Exploring food safety knowledge and practices in Lebanon	[97]	No results for urban LMIC
Knowledge and Perception about Food Adulteration Problem among School Children in Bangladesh	[98]	Full text paper unavailable
Food safety knowledge, attitudes and practices among consumers in developing countries: An international survey	[99]	No results for urban LMIC
Food safety knowledge among Jordanians: A national study	[100]	No results for urban LMIC
Food hygiene knowledge, attitudes and practices among students at the Cheikh Anta Diop University of Dakar (UCAD) Senegal	[101]	Full text paper unavailable

Food safety knowledge, attitudes and practices of mothers - findings from focus group studies in South India	[102]	No results for urban LMIC
Food Safety Knowledge, Attitudes, Practices, and Associated Factors Among Adult Consumers in Bangladesh: Findings From a Nationwide Cross-Sectional Survey	[103]	No results for urban LMIC
Food Safety Consciousness and Consumers◎Milk Purchasing Behavior: Evidence from a Developing Country	[104]	No consumer KAP focus
Assessment of food safety knowledge among households in Akwa Ibom State, Nigeria	[105]	No results for urban LMIC
Shifts in Food Consumption Practices among Middle-Class Households in Bengaluru India	[106]	COVID-19 study
Knowledge, attitude and practices among parents regarding food poisoning: a cross-sectional study from Palestine	[107]	No results for urban LMIC
Toxoplasmosis-Related Knowledge and Preventive Practices among Undergraduate Female Students in Jordan	[108]	No results for urban LMIC
Assessment of mothers◎knowledge and practice towards aflatoxin contamination in complementary foods in Ethiopia: From pre-harvest to household	[109]	No results for urban LMIC
Assessment of consumer◎ knowledge and practices on pesticide residues reduction in tomatoes in Mvomero, Morogoro region, Tanzania	[110]	No results for urban LMIC
Survey of knowledge, and attitudes to storage practices preempting the occurrence of filamentous fungi and mycotoxins in some Ghanaian staple foods and processed products	[111]	No results for urban LMIC
Assessment of community knowledge, attitude and practice on milk borne zoonoses disease in Debre-Birhan town, north Shewa, Ethiopia	[112]	No consumer KAP focus
Knowledge, attitude, and practices concerning presence of molds in foods among members of the general public in Malawi	[113]	No results for urban LMIC
◎We dry contaminated meat to make it safe◎ An assessment of knowledge, attitude and practices on anthrax during an outbreak, Kisumu, Kenya, 2019	[114]	No results for urban LMIC

Food hygiene practices and its associated factors among model and non model households in Abobo district, southwestern Ethiopia: Comparative cross-sectional study	[115]	No results for urban LMIC
Consumers◎knowledge regarding antibiotic residues and their consciousness about food safety issues in livestock products	[116]	No results for urban LMIC
Awareness and attitudes towards anthrax and meat consumption practices among affected communities in Zambia: A mixed methods approach	[117]	No results for urban LMIC
Perception and adoption of food safety practices (FSP) among beef sellers and consumers: Empirical evidence from Ghana	[118]	No consumer KAP focus
A study on knowledge and preventive practices related to avian influenza among higher secondary school students of Rajbiraj municipality, Nepal	[119]	Not foodborne illness
The knowledge, attitudes, and practices toward food additives in personnel of Isfahan University of Medical Sciences in Iran	[120]	Not low income and lower-middle income country
Food Safety Knowledge and Practices of Male Adolescents in West of Iran	[121]	Not low income and lower-middle income country
The knowledge, attitude, and practices of secondary high school students regarding food safety and hygiene in Khorramdarreh, Iran	[122]	Not low income and lower-middle income country

Table S3: PRISMA 2020 checklist.

Section and Topic	Item #	Checklist item	Location where item is reported
**TITLE**			
Title	1	Identify the report as a scoping review.	Title
**ABSTRACT**			
Abstract	2	See the PRISMA 2020 for Abstracts checklist.	Abstract
**INTRODUCTION**			
Rationale	3	Describe the rationale for the review in the context of existing knowledge.	Introduction
Objec-tives	4	Provide an explicit statement of the objective(s) or question(s) the review addresses.	Introduction
**METHODS**			
Eligibility criteria	5	Specify the inclusion and exclusion criteria for the review and how studies were grouped for the syntheses.	2.3
Infor-mation sources	6	Specify all databases, registers, websites, organisations, refer-ence lists and other sources searched or consulted to identify studies. Specify the date when each source was last searched or consulted.	2.2
Search strategy	7	Present the full search strategies for all databases, registers and websites, including any filters and limits used.	2.2
Selection process	8	Specify the methods used to decide whether a study met the in-clusion criteria of the review, including how many reviewers screened each record and each report retrieved, whether they worked independently, and if applicable, details of automation tools used in the process.	2.4
Data col-lection process	9	Specify the methods used to collect data from reports, including how many reviewers collected data from each report, whether they worked independently, any processes for obtaining or con-firming data from study investigators, and if applicable, details of automation tools used in the process.	2.5
Data items	10a	List and define all outcomes for which data were sought. Specify whether all results that were compatible with each outcome do-main in each study were sought (e.g. for all measures, time points, analyses), and if not, the methods used to decide which results to collect.	2.5
	10b	List and define all other variables for which data were sought (e.g. participant and intervention characteristics, funding sources). De-scribe any assumptions made about any missing or unclear	2.5

Section and Topic	Item #	Checklist item	Location where item is reported
		information.	
Study risk of bias assessment	11	Specify the methods used to assess risk of bias in the included studies, including details of the tool(s) used, how many reviewers assessed each study and whether they worked independently, and if applicable, details of automation tools used in the process.	2.6
Effect measures	12	Specify for each outcome the effect measure(s) (e.g. risk ratio, mean difference) used in the synthesis or presentation of results.	NA
Synthesis methods	13a	Describe the processes used to decide which studies were eligible for each synthesis (e.g. tabulating the study intervention characteristics and comparing against the planned groups for each synthesis (item #5)).	2.1 and 2.3
	13b	Describe any methods required to prepare the data for presentation or synthesis, such as handling of missing summary statistics, or data conversions.	2.5
	13c	Describe any methods used to tabulate or visually display results of individual studies and syntheses.	2.5
	13d	Describe any methods used to synthesize results and provide a rationale for the choice(s). If meta-analysis was performed, describe the model(s), method(s) to identify the presence and extent of statistical heterogeneity, and software package(s) used.	2.1 (meta-analysis was not performed)
	13e	Describe any methods used to explore possible causes of heterogeneity among study results (e.g. subgroup analysis, meta-regression).	NA
	13f	Describe any sensitivity analyses conducted to assess robustness of the synthesized results.	NA
Reporting bias assessment	14	Describe any methods used to assess risk of bias due to missing results in a synthesis (arising from reporting biases).	NA
Certainty assessment	15	Describe any methods used to assess certainty (or confidence) in the body of evidence for an outcome.	NA
**RESULTS**			
Study selection	16a	Describe the results of the search and selection process, from the number of records identified in the search to the number of studies included in the review, ideally using a flow diagram.	Figure 1
	16b	Cite studies that might appear to meet the inclusion criteria, but which were excluded, and explain why they were excluded.	Figure 1

Section and Topic	Item #	Checklist item	Location where item is reported
Study charac-teristics	17	Cite each included study and present its characteristics.	Table 2
Risk of bias in studies	18	Present assessments of risk of bias for each included study.	Table 3
Results of individ-ual stud-ies	19	For all outcomes, present, for each study: (a) summary statistics for each group (where appropriate) and (b) an effect estimate and its precision (e.g. confidence/credible interval), ideally using struc-tured tables or plots.	Table 2
Results of synthe-ses	20a	For each synthesis, briefly summarise the characteristics and risk of bias among contributing studies.	3.6 and 3.7
	20b	Present results of all statistical syntheses conducted. If meta-analysis was done, present for each the summary estimate and its precision (e.g. confidence/credible interval) and measures of statistical heterogeneity. If comparing groups, describe the direc-tion of the effect.	3.6 and 3.7
	20c	Present results of all investigations of possible causes of hetero-geneity among study results.	3.6 and 3.7
	20d	Present results of all sensitivity analyses conducted to assess the robustness of the synthesized results.	3.6 and 3.7
Reporting biases	21	Present assessments of risk of bias due to missing results (arising from reporting biases) for each synthesis assessed.	3.3
Certainty of evi-dence	22	Present assessments of certainty (or confidence) in the body of evidence for each outcome assessed.	NA
**DISCUSSION**			
Discus-sion	23a	Provide a general interpretation of the results in the context of other evidence.	Yes
	23b	Discuss any limitations of the evidence included in the review.	Yes
	23c	Discuss any limitations of the review processes used.	Yes
	23d	Discuss implications of the results for practice, policy, and future research.	Yes
**OTHER INFORMATION**			
Registra-tion and	24a	Provide registration information for the review, including register name and registration number, or state that the review was not	The review protocol was registered and published on Open Science Framework

Section and Topic	Item #	Checklist item	Location where item is reported
protocol		registered.	(OSF): https://doi.org/10.17605/OSF.IO/HBYJ6
	24b	Indicate where the review protocol can be accessed, or state that a protocol was not prepared.	Open Science Framework (OSF)
	24c	Describe and explain any amendments to information provided at registration or in the protocol.	NA
Support	25	Describe sources of financial or non-financial support for the review, and the role of the funders or sponsors in the review.	Funding sources
Competing interests	26	Declare any competing interests of review authors.	Conflicts of Interest
Availability of data, code and other materials	27	Report which of the following are publicly available and where they can be found: template data collection forms; data extracted from included studies; data used for all analyses; analytic code; any other materials used in the review.	Data availability

From: Page MJ, McKenzie JE, Bossuyt PM, Boutron I, Hoffmann TC, Mulrow CD, et al. The PRISMA 2020 statement: an updated guideline for reporting systematic reviews. BMJ 2021;372:n71. doi: 10.1136/bmj.n71. This work is licensed under CC BY 4.0. To view a copy of this license, visit https://creativecommons.org/licenses/by/4.0/
